# Efficacy of Canaloplasty for the Management of Primary Open-Angle Glaucoma: Protocol for a Systematic Review

**DOI:** 10.2196/69527

**Published:** 2025-10-14

**Authors:** Mohammed Ma'arij Anwar, Sobia Iqbal, Ahmed Osman, Celia Alcalde, Andrew Tatham

**Affiliations:** 1 Edinburgh Medical School University of Edinburgh Edinburgh United Kingdom; 2 Faculty of Engineering Royal School of Mines Imperial College London London United Kingdom; 3 Edinburgh Royal Infirmary Edinburgh United Kingdom; 4 Faculty of Medicine Imperial College London London United Kingdom; 5 Princess Alexandra Eye Pavilion Edinburgh United Kingdom

**Keywords:** systematic review, ab interno canaloplasty, glaucoma, minimally invasive surgery, meta-analysis, protocol

## Abstract

**Background:**

Glaucoma forms the leading cause of irreversible blindness worldwide, with a disproportionately rising prevalence in Asian and African countries. Primary open-angle glaucoma (POAG) accounts for the majority of cases. Medical therapies for POAG are not without side effects, and surgical treatments carry high complication rates. Ab interno canaloplasty promises a safer, minimally invasive, yet effective treatment option for mild-to-moderate POAG, as well as a cost-effective technique for low-resource countries. However, no systematic review currently exists to verify this procedure’s efficacy.

**Objective:**

The aim of this study was to develop a protocol for a systematic review aimed at evaluating the efficacy of canaloplasty against all other forms of POAG treatment.

**Methods:**

This systematic review and meta-analysis will include randomized controlled trials evaluating the short-term, medium-term, and long-term efficacy and safety of ab interno canaloplasty in treating POAG in comparison to all other treatments. Mean changes in intraocular pressure will form the primary outcome measure. Secondary outcome measures include proportion of participants who are medication-free after treatment and mean changes in the health-related quality of life. MEDLINE, Embase, Cochrane Library, and ClinicalTrials.gov databases will be searched for relevant randomized controlled trials. All studies will be subject to prespecified inclusion and exclusion criteria. The quality of the eligible randomized controlled trials will be assessed using the Cochrane risk of bias tool. Data will be extracted with a focus on raw data where possible, and analysis will be performed using RevMan 5.4 software to compare the mean changes in intraocular pressure (mm Hg) between ab interno canaloplasty and other comparator therapies. A funnel plot will be used to assess the risk of publication bias if 10 or more trials are included in the review. *I*^2^ statistics will be used to assess heterogeneity. Sensitivity analysis will be conducted to exclude studies with a high risk of bias and, where possible, on the primary outcome. The GRADE (Grading of Recommendations, Assessment, Development, and Evaluation) approach will be used to summarize the main findings.

**Results:**

The results of this systematic review are not yet available as it is still at the protocol stage. This protocol was registered on the PROSPERO database for systematic reviews (CRD42024558671) on June 27, 2024. Data collection for this review began on July 14, 2024, with the anticipated completion date being early 2026.

**Conclusions:**

The findings will be important to patients, clinicians, and policymakers worldwide in addressing the growing burden and health inequality of glaucoma.

**Trial Registration:**

PROSPERO CRD42024558671; https://www.crd.york.ac.uk/PROSPERO/view/CRD42024558671

**International Registered Report Identifier (IRRID):**

PRR1-10.2196/69527

## Introduction

Glaucoma is an umbrella term used to describe a group of chronic progressive optic neuropathies associated with raised intraocular pressure (IOP) [1]. It forms the leading cause of irreversible blindness worldwide, contributing to 11% of all blindness in adults aged 50 years and older in 2020 [2]. Glaucoma currently affects 76 million adults around the globe, and this is predicted to rise to 111.8 million by 2040, with primary open-angle glaucoma (POAG) being the common subtype [3]. Furthermore, this rising prevalence is disproportionately affecting Asian and African populations, with, for instance, a higher incidence and severity of the disease being found in younger adults in Nigeria [4], which can be attributed to poor access to treatment [4]. It is therefore of interest to adopt new, cost-effective yet efficient methods of treatment that may be readily delivered in low-resource countries to ease the rising epidemic of glaucoma. This is not only vital to providing care in low-resource countries most affected by glaucoma and tackling the health inequality but would be of use at a time of increasing health care costs in the United Kingdom [5].

The treatment and prevention of glaucoma progression and thereby preservation of vision is centered around reducing IOP with the regular use of topical eyedrops, laser therapy, and/or surgery [6]. Unfortunately, intolerance of side effects such as dry eye and irritation has resulted in low adherence among patients using eyedrops in comparison to adherence to medications for other chronic conditions [7,8]. Surgical techniques such as the gold-standard trabeculectomy are not without significant risks, with a 78% increase in cataract formation and up to 21.3% of patients requiring complication-related surgery [9,10]. Surgery is therefore reserved for cases refractory to other therapies [11].

Minimally invasive surgical procedures show the potential to effectively reduce IOP in the long term, removing the need for indefinite daily self-administration of eye drops. This may not only be preferable for patients but also prevents vision loss related to nonadherence to topical treatment.

Ab interno canaloplasty is a new, minimally invasive surgical procedure regarded as highly effective in reducing IOP, with fewer complications and a simpler postoperative care regimen than other surgical interventions [12,13]. It acts to permanently dilate Schlemm’s canal, which facilitates up to 96% of aqueous humour drainage and is found to be of reduced dimensions in glaucomatous eyes [14,15]. It is currently approved by NICE (National Institute for Health and Care Excellence) for cases of mild-to-moderate POAG [16]. Ab interno canaloplasty promises a safer and more cost-effective alternative to other treatment options in the long term. However, although NICE acknowledges the procedure as safe, there is little evidence on its efficacy [16]. Considering the potential benefits for patients worldwide and the recent uptake of the procedure in the United Kingdom, it is important to critically evaluate the evidence for whether canaloplasty is both efficacious and cost-effective. No systematic review currently exists to consolidate and verify this procedure’s efficacy.

This systematic review aims to compare the following.

1. The short-term and long-term efficacy of canaloplasty against all other forms of open-angle glaucoma treatment.
2. The mean IOP changes in canaloplasty to determine the therapeutic effects.
3. The safety of canaloplasty as a treatment against other treatment options for open-angle glaucoma.

## Methods

### Study Design

We will conduct a systematic review and meta-analysis of randomized controlled trials performing canaloplasty. This protocol will be conducted in accordance with the PRISMA-P (Preferred Reporting Items for Systematic Reviews and Meta-analyses Protocol) statement (Multimedia Appendix 1) and has been prospectively registered on PROSPERO (registration: CRD42024558671). All randomized controlled trials will be included, irrespective of their publication status or language. If studies are not in English, we will use translation assistance (other reviewers who are native to the language or Google PDF translator software).

### Types of Participants

Participants included will be those with POAG. We will also include all participants with ocular hypertension, normal-tension glaucoma, or possible glaucoma (suspects for glaucoma). There will be no restrictions regarding region, nation, sex, duration, setting, or demographic factors.

### Types of Interventions

We will consider ab interno canaloplasty performed with the OMNI Surgical System or the iTRACK microcatheter. However, we will not apply any particular inclusion or exclusion criteria to these or other treatment delivery parameters. Ab interno canaloplasty will be compared against any comparator, including the following: (1) conventional glaucoma surgery (trabeculectomy), (2) laser treatment (trabeculoplasty) (3) other minimally invasive glaucoma techniques, and (4) medical treatment.

### Types of Outcome Measures

Our primary outcome will be the mean change in IOP measured using the Goldmann applanation tonometry. However, the reporting of a particular outcome as a criterion is not necessary for eligibility for review nor will we exclude studies solely on an outcome of interest not being included.

### Secondary Outcomes

The secondary outcomes will be as follows: (1) proportion of participants who were medication-free (not using eyedrops) after canaloplasty; (2) mean change in the number of IOP‐lowering topical eyedrops taken per day; (3) proportion of participants who required further glaucoma surgery, as recorded by the investigators of the included trial; (4) rate of visual field progression (decibels/time) or proportion of participants whose field loss progressed in the follow‐up period; (5) mean change in the health-related quality of life; and (6) cost effectiveness reporting for each intervention. The Health-Related Quality of Life Scale [17] will be used in our study.

### Adverse Effects

The adverse effects reported are as follows: (1) loss of visual acuity (more than 2 Snellen lines or more than 0.3 logMAR, according to the method of recording visual acuity) or loss of light perception; (2) bleeding, as recorded by the investigators; (3) endophthalmitis, as recorded by the investigators; and (4) IOP spikes (postoperative rise in IOP, measured using Goldmann applanation tonometry, of more than 10 mm Hg compared to the previous assessment, including measurements taken during the first postoperative month).

### Search Methods for the Identification of Studies

#### Electronic Searches

We will search the following electronic databases for randomized controlled trials, placing no restrictions to language or year of publication: (1) Cochrane Central Register of Controlled Trials (which contains the Cochrane Eyes and Vision Trials Register) in the Cochrane Library (February 2024), (2) MEDLINE Ovid (1946 to present), (3) Embase Ovid (1980 to present), (4) International Standard Randomized Controlled Trial Number registry, (5) US National Institutes of Health Ongoing Trials Register ClinicalTrials.gov, and (6) World Health Organization (WHO) International Clinical Trials Registry Platform.

Preliminary searches of the databases yielded 4000 papers that met the search criteria and will be reviewed, although this number may increase by the time we conduct this study. As we are placing no language restrictions on the systematic reviews, we aim to translate and extract data by using the Google PDF translator as well as our diverse authors who possess professional proficiency in English, Urdu, Hindi, Spanish, and Arabic.

#### Searching Other Resources

To identify further studies that may meet the inclusion criteria, we will search the reference lists of all the included studies. We will also search conference proceedings from the following meetings from 1980 onwards: (1) American Academy of Ophthalmology, (2) Association of Research in Visual Science and Ophthalmology, (3) World Ophthalmology Congress, and (4) World Glaucoma Congress.

### Data Collection and Analysis

#### Selection of Studies

We will remove duplicate references and import the search results into the web-based review management software Covidence. Two review authors (MMA and SI) will independently screen the titles and abstracts for all the studies identified by the search. If abstracts are not available, a full-text screen of the study will be performed. We will then retrieve full‐text reports of any potential studies for inclusion to assess eligibility. In the event of any disagreement regarding eligibility, a third review author (AT) will be the arbitrator. If we reject any full text reports, we plan to record the reasons for this. If we have doubts about either the study population or the intervention, we will contact the study authors (via email) for clarification so that the decision to include or exclude can be properly informed. We will give them a month to reply.

#### Data Extraction and Management

In situations where information from the included studies is missing or unclear, our protocol will involve reaching out to the respective individuals or organizations for clarification. We aim to acquire the most comprehensive numerical data accessible to enhance the analysis of the included studies. Rather than opting for less accurate approaches such as extracting numeric values from graphs, our plan will be to gather the data directly from individuals or organizations. In the event that extraction from graphs becomes necessary, 2 review authors will independently perform the task, with a third review author serving as an arbitrator in case of any discrepancies.

#### Risk of Bias Assessment in the Included Studies

Two authors (MMA and SI) will independently assess the studies for risk of bias by using the Cochrane risk of bias tool [18]. We will evaluate the 7 domains, reporting bias as low, high, or unclear.

#### Measures of Treatment Effect

Continuous data will be summarized as the mean difference or standardized mean difference, with a 95% CI comparing canaloplasty with the comparator group. We will express dichotomous data as risk ratios with 95% CIs.

#### Dealing With Missing Data

For studies with missing data, we will contact study investigators by email and give them 1 month to respond. Ideally, the studies we review will have used intention-to-treat analyses. We will document the proportion of missing data within each trial. We will attempt to assess the reasons for missing data to determine whether they are missing at random. If we consider that they are missing at random, we will use the available data.

#### Reporting Bias Assessment

A funnel plot will be used to assess the risk of publication bias if 10 or more trials are included in the review. Where appropriate, we will perform statistical tests for funnel plot asymmetry to assess publication bias, as recommended in chapter 13 of the Cochrane Handbook for Systematic Reviews of Interventions [19].

#### Synthesis Methods

Data analysis will be performed using RevMan software (version 5.4; Cochrane). We will report funnel plots for bias and risk ratios and synthesize primary outcomes with descriptive statistics as well as funnel plots where appropriate.

#### Investigation of Heterogeneity and Subgroup Analysis

We plan to assess the heterogeneity between trials by assessing forest plots and examining the *I*^2^ value and 95% CIs. We will examine the *I*^2^ value and use the guidance provided by the  Cochrane Handbook for Systematic Reviews of Interventions  for interpretation of this value [18].

#### Sensitivity Analysis

We will conduct a sensitivity analysis, which will exclude studies that are classified as having overall high risk of bias in one or more key domains. Where possible, we will also perform the following sensitivity analyses for our primary outcome: (1) inclusion of only trials with a low risk of attrition bias, (2) inclusion of trials with a total sample size of 50 or more randomized participants to detect potential small‐study effects, and (3) inclusion of mixed types of glaucoma in the study population.

#### Certainty of the Evidence Assessment

We will summarize the main findings, including strengths and limitations of evidence for both primary and secondary outcomes, using the GRADE (Grading of Recommendations, Assessment, Development, and Evaluation) approach [20]. We plan to provide a summary of the effectiveness of the intervention and general interpretation of the evidence in the context of other evidences and implications for practice and future research. We will also present a “Summary of findings” table for each comparison listed in the “Types of Interventions” for each outcome.

## Results

This review aims to evaluate the short-term, medium-term, and long-term efficacy of ab interno canaloplasty in treating patients with suspected or confirmed POAG in comparison to other glaucoma treatments. The results of this review are not yet available, as it is still at the protocol stage. This protocol was registered on the PROSPERO database for systematic reviews (CRD42024558671) on June 27, 2024. Data collection for this review began on July 14, 2024. The anticipated completion date of this review is early 2026. The findings of this review will help verify this procedure’s efficacy, which will aid in its adoption worldwide, thereby reducing the level of health inequality and the global burden imposed by this condition.

## Discussion

### Canaloplasty Versus Current Glaucoma Treatments

Ab interno canaloplasty is an exciting new treatment that, despite its minimally invasive nature, offers promising results with fewer complications and a simpler postoperative care regimen than other surgical interventions [12,13]. It acts to permanently dilate the Schlemm’s canal, which is found to be of reduced dimensions in glaucomatous eyes [14,15]. Ab interno canaloplasty is currently approved by NICE for cases of mild-to-moderate POAG [16]; however, there is little evidence on its efficacy [16]. Considering the potential benefits for patients worldwide, it is important to critically evaluate the evidence for whether canaloplasty is both efficacious and cost-effective. To date, no systematic review has been conducted on the efficacy of canaloplasty. Trabeculotomy remains the gold standard in the treatment of glaucoma.

### Comparison With Prior Work

Previous reviews of glaucoma treatment document large heterogeneities with a variety of descriptions for IOP and safety outcomes [21]. This can lead to research resource wastage and reduce the quality of strength of glaucoma studies. IOP is the most clinically relevant and consistent end point in glaucoma management and the most frequently used among studies evaluating surgical success. This will improve the validity of the analysis. Furthermore, to ensure that the reporting is standardized with previous evidence of glaucoma care [1], we will report outcomes in the short term (6-18 months), medium term (18-36 months), and long term (36 months or longer). This will also mitigate any variation in the follow-up durations across studies, which are likely to be similar due to the novel nature of the procedure. In the circumstance that the variation is high, we will address this further through subanalysis. Lastly, including health-related quality of life and the proportion of patients remaining medication-free after surgery as secondary outcomes will also complement the IOP measurements to assess surgical success. Due to differences in the technique, notably between OMNI and iTrack, with the former involving both canaloplasty and goniotomy in one procedure and the latter comprising of injecting more viscoelastic fluid inside of Schlemm’s canal with potential added efficacy, a subanalysis will also be conducted in the treatment outcomes of the primary and secondary aims to highlight any clinical differences or benefits.

Another systematic review on the primary outcomes of glaucoma care suggested visual field acuity, safety, and optic nerve head morphology as the most important outcomes to glaucoma experts and treatment [22]. In an effort to standardize the reporting of outcomes and strengthen the research, we hope to report mean changes of IOP as our primary outcome, as this is the most reported result in primary studies and most used and important primary outcome [23].

### Conclusions

Randomized controlled trials involving eyes can be difficult to conduct. To conduct the most comprehensive review, we plan to exclude within-person studies because of the potential for medical treatment such as 1-eye treatment affecting the other eye results. For the same reason, we will exclude paired‐eye studies, which randomize 1 eye to 1 intervention and the other to the alternative intervention. We will however review studies, which include 1 eye per participant. If a study randomized eyes, regardless of whether they were in the same participant, we will contact the study authors for more information. If possible, we will only include eyes that had the first intervention.

## Appendix

Multimedia Appendix 1 PRISMA-P checklist.

## Abbreviations

GRADE: Grading of Recommendations, Assessment, Development, and Evaluation
IOP: intraocular pressure
NICE: National Institute for Health and Care Excellence
POAG: primary open-angle glaucoma
PRISMA-P: Preferred Reporting Items for Systematic Reviews and Meta-analyses Protocols
WHO: World Health Organization

## References

[1] Weinreb RN, Aung T, Medeiros FA (2014). The pathophysiology and treatment of glaucoma: a review. JAMA.

[2] Sun Y, Chen A, Zou M, Zhang Y, Jin L, Li Y, Zheng D, Jin G, Congdon N (2022). Time trends, associations and prevalence of blindness and vision loss due to glaucoma: an analysis of observational data from the Global Burden of Disease Study 2017. BMJ Open.

[3] Tham Y, Li X, Wong TY, Quigley HA, Aung T, Cheng C (2014). Global prevalence of glaucoma and projections of glaucoma burden through 2040: a systematic review and meta-analysis. Ophthalmology.

[4] Allison Karen, Patel Deepkumar, Alabi Omobolanle (2020). Epidemiology of glaucoma: the past, present, and predictions for the future. Cureus.

[5] Health funding data analysis. BMA.

[6] Schuster AK, Erb C, Hoffmann EM, Dietlein T, Pfeiffer N (2020). The diagnosis and treatment of glaucoma. Dtsch Arztebl Int.

[7] Economou MA, Laukeland HK, Grabska-Liberek I, Rouland J (2018). Better tolerance of preservative-free latanoprost compared to preserved glaucoma eye drops: the 12-month real-life FREE study. Clin Ophthalmol.

[8] Tapply I, Broadway DC (2021). Improving adherence to topical medication in patients with glaucoma. Patient Prefer Adherence.

[9] Olayanju JA, Hassan MB, Hodge DO, Khanna CL (2015). Trabeculectomy-related complications in Olmsted County, Minnesota, 1985 through 2010. JAMA Ophthalmol.

[10] Winuntamalakul Y, Chansangpetch S, Ratanawongphaibul K, Itthipanichpong R, Manassakorn A, Tantisevi V, Rojanapongpun P (2023). Two-year outcomes of trabeculectomy and phacotrabeculectomy in primary open angle versus primary angle closure glaucoma. J Glaucoma.

[11] King A, Azuara-Blanco A, Tuulonen A (2013). Glaucoma. BMJ.

[12] Khaimi MA (2015). Canaloplasty: a minimally invasive and maximally effective glaucoma treatment. J Ophthalmol.

[13] Riva I, Brusini P, Oddone F, Michelessi M, Weinreb RN, Quaranta L (2019). Canaloplasty in the treatment of open-angle glaucoma: a review of patient selection and outcomes. Adv Ther.

[14] Lewczuk K, Jabłońska J, Konopińska J, Mariak Z, Rękas M (2022). Schlemm's canal: the outflow 'vessel'. Acta Ophthalmol.

[15] Allingham RR, de Kater AW, Ethier CR (1996). Schlemm's canal and primary open angle glaucoma: correlation between Schlemm's canal dimensions and outflow facility. Exp Eye Res.

[16] (2022). Ab interno canaloplasty for open-angle glaucoma. NICE.

[17] Health-related quality of life scale. SPARQTools.

[18] Higgins J, Savovi J, Page M, Elbers R, Sterne J (2019). Assessing risk of bias in a randomized trial. Cochrane Handbook for Systematic Reviews of Interventions Version 6.4 (updated August 2023).

[19] Page M, Higgins J, Sterne J (2019). Assessing risk of bias due to missing results in a synthesis. Cochrane Handbook for Systematic Reviews of Interventions Version 6.4 (updated August 2023).

[20] GRADEpro Guideline Development Tool.

[21] Ismail R, Azuara-Blanco A, Ramsay CR (2015). Outcome measures in glaucoma: a systematic review of Cochrane reviews and protocols. J Glaucoma.

[22] Hu K, Vemulapalli K, Gandhewar R, Shah A, Virgili G, Bunce C, Gazzard G (2022). Minimally invasive trabecular meshwork surgery for open-angle glaucoma. Cochrane Database of Systematic Reviews.

[23] Ismail R, Azuara-Blanco A, Ramsay CR (2016). Consensus on outcome measures for glaucoma effectiveness trials: results from a Delphi and nominal group technique approaches. J Glaucoma.

